# Grass in Rum

**Published:** 1854-06

**Authors:** 


GRASS IN RUM.—An old fellow in Missouri, who was in the habit of "not belonging to the Temperance Society," was in the act of taking a nip one day before a young Virginian.

"What do you drink?" asked the latter.

"Brandy and water," was the reply.

"Why don't you drink mint juleps?"

"Mint juleps?" queried the old man, "why, what in the name of drink is that?"

"A most delicious drink," was the answer; "and I'll show you how to make it, as I see you have mint growing almost at your door."

The young fellow soon produced the juleps, and the old man was delighted with it.

About a month after, on his return home, the Virginian thought he would stop at his old friend's and "indulge," but judge of his surprise when his inquiries at the door for his friend was answered by an aged female darkey, with:

"Oh, Massa's dead and gone dis two weeks!"

"Dead!" exclaimed the young man, "why, how strange! What did he die of?"

"Oh, I d'no," returned the woman, "only a fellow come along about a monf ago and larnt him to drink *grass in he rum*, and it killed him in two weeks."

---

Love that has nothing but beauty to keep it in good health, is short-lived, and apt to have ague fits.

The parent who would train up a child in the way he should go, must go in the way he would train up his child

